# Severe neonatal hyperbilirubinemia secondary to combined RhC hemolytic disease, congenital hypothyroidism and large adrenal hematoma: a case report

**DOI:** 10.1186/s12887-022-03594-7

**Published:** 2022-09-11

**Authors:** Chengiun Dai, Chun Chen, Liqiong Jiang, Yilin Zhu, Chunlin Wang

**Affiliations:** Department of Pediatrics, The First Affiliated Hospital, College of Medicine, Zhejiang University, Hangzhou, 310003 Zhejiang Province China

**Keywords:** Neonatal hyperbilirubinemia, RhC hemolytic disease, Congenital hypothyroidism, Adrenal hemorrhage, Case report

## Abstract

**Background:**

ABO blood group incompatibility, neonatal sepsis, G-6-PD deficiency, thyroid dysfunction, and hereditary spherocytosis are all probable causes of neonatal hyperbilirubinemia. However, the etiology of some hyperbilirubinemia is extremely complicated, which may be caused by multiple factors, resulting in severe jaundice. We report a case of severe jaundice due to three causes, showing the significance for the investigation of the etiology of neonatal hyperbilirubinemia.

**Case presentation:**

At 96 h of life, a full-term and vaginal delivery male infant with yellowish discoloration of body was transferred to our hospital. When he entered neonatal intensive care unit on the fourth day after birth, he developed jaundice and the transcutaneous bilirubin was 28 mg/dl. Total bilirubin was 540.2 μmol/L, while the indirect bilirubin was 516.7 μmol/L. Both parents and the baby’s blood types were O Rh(D +), and direct coomb’s test was negative. But mother’s indirect coomb’s test was positive. Investigating for minor blood group revealed that the father’s blood type of Rh was CCDee, the mather’s was ccDEE, and CcDEe for the baby. After intensive phototherapy and double volume exchange transfusion, the total bilirubin remained at 303 μmol/L. At day 10, the bilirubin level was 303.5 μmol/L, intensive phototherapy was continued, and intravenous immunoglobulin was used again. The test for thyroid hormones at day 10, the TSH was 13.334mIU/L. And the screening for congenital hypothyroidism showed the TSH was 33mIU/L. Because of the palpable abdominal mass, ultrasound and MRI was done, showed a huge mass in the right adrenal gland. Brainstem auditory evoked potential was performed at day 7, which indicated hearing impairment (65db for left ear and 70db for the right). Euthyrox and intermittent phototherapy were given as following treatment. The jaundice did not subside until the 12th day.

**Conclusion:**

Even if their parents' ABO blood group and Rh (d) are consistent, a Coomb test is required for newborns with hyperbilirubinemia since they may have minor blood group incompatibilities. When bilirubin rises rapidly or the clinical treatment effect is inadequate, additional causes should be aggressively screened. Adrenal ultrasound should be performed on newborns with palpable abdominal mass, anemia and jaundice to determine whether there is adrenal hemorrhage.

## Background

Neonatal hyperbilirubinemia is the most widespread disease in the neonatal period. Hemolytic disease of the newborn(HDN) is a prevalent cause of severe hyperbilirubinemia that frequently results in severe jaundice, the prevalence rate is about 12.9% [1]. Alloimmune HDN predominantly affects the Rhesus (Rh), A, B, AB, and O blood groups, whereas minor blood group incompatibilities (Kell, Duffy, MNS, P, and Diego systems) can also cause irreversible damage. About 3–5% cases of HDN were reported caused by minor blood group incompatibility [2]. Congenital hypothyroidism(CHT) is another cause of hyperbilirubinemia, yet it usually leads to mild to moderate jaundice and persistent jaundice. There are few reports of severe jaundice induced by CH [3], the prevalence rate is about 0.3% [1]. Adrenal hemorrhage in newborn infants is an uncommon occurrence that can cause jaundice duo to extravascular hemolysis, with the incidence proportion of about 0.17%—0.3% [4]. A case of special hyperbilirubinemia coupled with RhC hemolytic disease, congenital hypothyroidism caused by DUOX2 gene mutation, and large adrenal hemorrhageare described.

## Case presentation

**Fig. 1:**
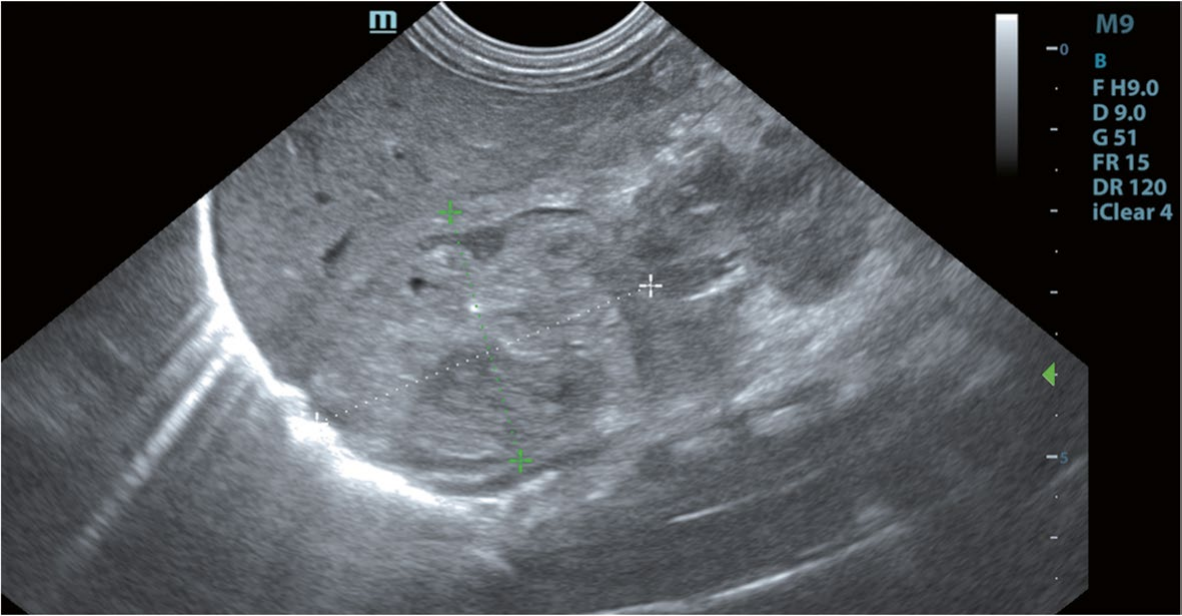
Abdominal ultrasound showed a huge mass in the right adrenal gland

**Fig. 2:**
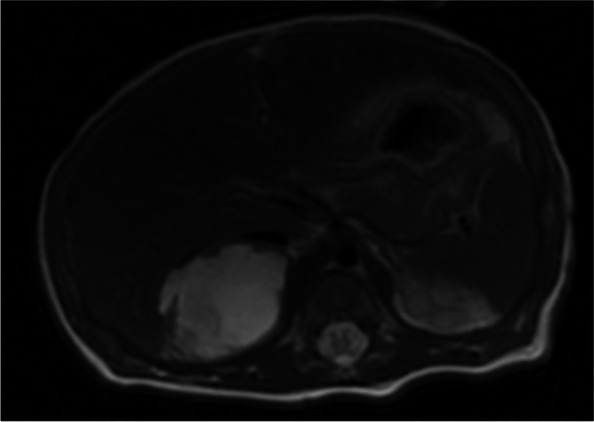
Abdominal MRI showed the adrenal hemorrhage(3.1*3.6 cm)

A full-term(40^+5^ weeks) male infant was transferred to our hospital at 96 h of life with yellowish discoloration of body. The boy was born by normal spontaneous vaginal delivery to a 32-year-old G2P1 woman. The birth weight was 3660 g, and APGAR scores were 9 and 10 at 5 and 10 min, respectively. The jaundice appeared at day 2 after birth, but his parents were unconcerned about it. The baby didn't react well when he came to the hospital. The transcutaneous bilirubin was 28 mg/dl when he was admitted to the neonatal intensive care unit(NICU). Total serum bilirubin was 540.2 μmol/L(31.5 mg/dl), while the indirect bilirubin was 516.7 μmol/L(30.2 mg/dl). Hemoglobin was 173 g/L, white blood cell count was 11.01*10^9/L, platelet count was 223*10^9/L, and red blood cell count was 4.74*10^12/L, according to a complete blood count. Reticulocytes made up 2.9% of the total. The blood types of both the parents and the baby’s were O Rh(D +), and direct coomb’s test was negative. However, mother’s indirect coomb’s test was positive, hence minor blood grouping and cross matching was implemented. Investigating for minor blood group revealed that the father’s blood type of Rh was CCDee, the mather’s was ccDEE, and CcDEe for the baby. Intensive phototherapy was initiated as soon as he entered NICU. After the blood types were confirmed, he underwent a double volume exchange transfusion. Intravenous fluids, intravenous immunoglobulin, penicilin and cefoperazone were among the other therapies. But until day 6, the total bilirubin was still 303 μmol/L(17.7 mg/dl), and rigorous phototherapy consistedcomprised of intravenous immunoglobulin to bind free antibody till day 10. Further tests revealed that glucose-6-phosphate dehydrogenase was 3693U/L, and the test for hereditary spherocytosis was negative. But thyroid hormone testing at day 10 revealed congenital hypothyroidism, with a TSH of 13.334mIU/L(normal:0.350–4.940mIU/L). And the screening for congenital hypothyroidism showed the TSH was 33mIU/L. Since a palpable abdominal mass was touched, abdominal ultrasound was done, and revealed a large lump in the right adrenal gland (Fig. 1). No obvious abnormality was discovered in head MRI, but the adrenal hemorrhage measuring 3.1*3.6 cm was demonstrated clearly in abdominal MRI (Fig. 2). Brainstem auditory evoked potential was executed at day 7, which showed hearing impairment( 65db for left ear and 70db for the right). Following treatment included euthyrox and intermittent phototherapy. The jaundice did maintained until the 12th day after birth. And the baby discharged from the hospital at day 13.

### Follow-up

Thyroid hormone was checked again 2 weeks following oral administration of euthyrox, and TSH was remained 16mIU/L. Hearing impairment has reappeared, according to brainstem auditory evoked potentials (25dbl for left ear and 40dbl for the right). Next generation sequencing revealed that the boy had compound heterozygous mutations in the DUOX2 gene, c.244 (exon 4) C > T and c.1883 (exon 16) delA, c.244 (exon 4) C > T heterozygous mutation was identified in promoter’s father, and c.1883 (exon 16) delA heterozygous mutation in his mather. At the 4th month follow-up visit, he was still taking euthalaz, but the adrenal ultrasound showed that the hematoma was almost completely absorbed.

## Discussion and conclusion

Neonatal hyperbilirubinemia is one of the most prevalent diseases in newborns, common causes include ABO blood group incompatibility, neonatal sepsis, G-6-PD deficiency, thyroid dysfunction, hereditary spherocytosis,etc. [3]. The majority of Rh blood group incompatibility is caused by anti-D, but a tiny portion is caused by other antibodies such as anti-C, anti-e, anti-c, etc. Minor blood group compatibilities, frequently lead to severe neonatal jaundice and hemolytic anemia, necessitating intensive phototherapy and even transfusion exchange. Although the invention of Rh immunoglobulin has considerably decreased the occurrence of RH anti-D hemolytic disease of newborn(HDN), hemolytic diseases caused by other uncommon antibodies remain difficult to ignore. In this case, the direct coombs test was negative, but the indirect coombs test was positive. Additionally, the presence of anti-C in the blood indicated the presence of Rh anti-C hemolysis. He had hazardous hyperbilirubinemia, but hemoglobin did not decrease dramatically, which was unusual in comparison to other cases: a case of neonatal hyperbilirubinemia induced by anti-C and anti-e was described by Deepak Sharma et al. The hemoglobin plummeted to 11 g/dl and the total blood bilirubin was 17.1 mg/dl at 48 h after birth, bilirubin decreased after intensive phototherapy [5]. Chen et al. reported a case of neonatal hemolysis stimulated by anti-C, anti-G, and anti-D. On the first day after birth, hemoglobin dropped to 13.9 g/dl and total bilirubin was 74.9 μmol/L [6]. In this situation, the reticulocytes and hemoglobin of the patient were still within the normal range, suggesting that the degree of hemolysis did not correspond to the degree of increased jaundice, and other factors were most likely to be combined.

Further examination confirmed our suspicion, congenital hypothyroidism is the second factor in his bilirubin increase. Thyroid hormones revealed that the TSH of baby was 13.334mIU/L, which increased to 16mIU/L over two weeks of oral administration of euthyroxine. An epidemiological survey in Iran conducted that hypothyroidism is responsible for around 4.2% of hyperbilirubinemia [7]. Although the large portion of CH manifestations are protracted jaundice, several large-sample studies have shown that congenital hypothyroidism can produce severe hyperbilirubinemia, prompting transfusion exchange [3]. Further next generation sequencing showed that there were compound heterozygous mutations in DUOX2 gene, which were c.244 (exon 4) C > T and c.1883 (exon 16) delA. The gene edits peroxidase enzyme, which is a key regulator of thyroxine synthesis. CH is interconnected to mutations in the DUOX2 gene in approximately 21%-45% [8]. The activity of uridine diphosphate glucuronyl transferase (UGT) in neonatal liver cells deminishes when there is a paucity of thyroxine. This is the main cause of hypothyroidism-induced jaundice. In contrast, newborns with congenital hypothyroidism, intestinal peristalsis slows down, and meconium excretion is delayed, augmenting the intestinal hepatic circulation of bilirubin and aggravating jaundice.

In addition to Anti C isoimmunization and CH, ultrasound of the adrenal glands and subsequent abdominal MRI reported that the child had a dominant right adrenal hemorrhage, which contributed to the overall rise in serum bilirubin. Neonatal adrenal hemorrhage is infrequent, with incidence proportion of about 0.17%—0.3%. Adrenal hemorrhage can occur in neonates owing to the relatively large size and distinctive vascularity, making them vulnerable to asphyxia, infection, birth injury, and abnormal blood coagulation. But we didn't find out the cause of adrenal hemorrhage in this case. As according statistics, roughly 70% of adrenal hemorrhages occur on the right side, which is compatible with our case. The neonatal adrenal hemorrhage may be asymptomatic, or exacerbate existing or persistent jaundice. Although some patients had scrotal hematoma and abdominal mass, most did not have adrenal insufficiency. Sáncheza et al. reported a newborn with ABO hemolytic disease and adrenal hemorrhage who developed jaundice. After enduring immunoglobulin and intensive phototherapy, he continued to have severe jaundice [9]. Another instance of hyperbilirubinemia with ABO hemolysis and adrenal hemorrhage was described by Eleonora tognato et al. Despite receiving immunoglobulin treatment, the patient required phototherapy until the 10th day after birth [10]. This is very similar to our patient. The efficacy of transfusion exchange, continuous phototherapy, and immunoglobulin application is not desirable in the first few days, which may be attached to the hematoma’s incessant extravascular hemolysis. During the follow-up, this hematoma was almost completely absorbed at the 4^th^ month.

This is the third example of hyperbilirubinemia with hemolytic disease and adrenal hematoma that has also been associated with congenital hypothyroidism to our knowledge. It was challenging to treat such a complicated jaundice. Our experience can potentially be used as a guide for future clinical practice.

For newborns with hyperbilirubinemia, Coomb test is required even if their parents' ABO blood group and Rh (d) are consistent, because they may have minor blood group incompatibilities. When bilirubin rises rapidly or the clinical treatment impact is insufficient, alternative factors, such as hypothyroidism, abnormal erythrocyte morphology, G-6-PD deficiency, etc., should be thoroughly investigated. Furthermore, when the newborn has severe jaundice or persistent jaundice, the pediatrician should pay attention to the abdominal physical examination. When there is a lump in the abdomen, adrenal ultrasound should be performed to see if there is any evidence of adrenal hemorrhage.

## Data Availability

The data supporting the conclusions of this article will be made available by the authors, without undue reservation.

## Abbreviations

CHT: Congenital hypothyroidism
HDN: Hemolytic disease of the newborn
NICU: Neonatal intensive care unit
UGT: Uridine diphosphate glucuronosyltransferase
